# Togetherness in a Safe and Motivating Environment at Meeting Places: Older Persons’ Experiences of Group Exercises

**DOI:** 10.1155/jare/6833782

**Published:** 2026-05-25

**Authors:** Daniella Dinse, Maria Haak, Ulrika Olsson Möller, Staffan Karlsson, Marie Nilsson

**Affiliations:** ^1^ Department of Nursing and Health Sciences, Faculty of Health Sciences, Kristianstad University, Kristianstad, Sweden, hkr.se

**Keywords:** health promotion interventions, meeting points, older adults, person-centredness, physical activity, senior centres

## Abstract

**Introduction:**

Many municipalities in Sweden offer group exercises for older persons at meeting places, i.e., senior centres. However, it is unclear whether older persons experience group exercises as in accordance with their needs and wishes.

**Aim:**

The aim was to explore older persons’ experiences of participating in group exercises held at meeting places in municipalities from a person‐centred perspective.

**Methods:**

Individual interviews were conducted with 18 persons aged 65 and over who participated in group exercises at meeting places in six different municipalities. The interview guide was based on a person‐centred framework. The data were analysed using content analysis, first deductively and then inductively.

**Findings:**

The findings were presented in two main categories. The first concerned incentives for older persons to continue exercising in groups, which were held at meeting places. Such incentives were the encouragement they felt from the power of the group and the fact that the group exercises made them feel good. They also felt a sense of worth from having time and effort invested in them. The second main category included mixed feelings about the interaction between the older persons and the stakeholders, primarily the exercise leader. On the one hand, the older persons felt a strong trust in the exercise leaders, but on the other, they felt ambivalent and had ambiguous feelings regarding their own opportunities to influence the group exercises.

**Conclusions:**

To conclude, the group exercises held at meeting places contribute to meaningful relationships and a sense of social belonging and togetherness. The intervention not only promotes physical and mental health but also counters loneliness and social isolation, and therefore needs to be scaled up even more. This study highlights the need to make group exercises more person‐centred and to introduce processes that include the needs and wishes of older persons in the design of such interventions.

## 1. Introduction

Physical activity is important for older persons. It brings many health benefits, promoting healthy ageing, maintaining independence, ensuring quality of life and increasing life expectancy [1–4]. Physical activity in groups improves social well‐being and motivates people to continue exercising [5, 6]. From a societal perspective, physical activity reduces the need for care and lowers social costs [7–9].

To achieve effects at an individual and societal level, physical activity needs to be part of the older persons’ everyday lives and to be sustained over time. Physical activity should also be accessible environmentally and economically [10]. Hence, the WHO [11] emphasises the importance for everyone, at a national level, to have access to supportive environments and diverse opportunities to be physically active in everyday life. Being able to be physically active in everyday life is an important part of active and healthy ageing. Important dimensions such as quality of life, emotional well‐being, social participation, functional ability and engagement in everyday activities are also important components of active and healthy ageing [4, 12]. As in all phases of one’s life, older persons are embedded in a social context in which the person and the environment are interrelated. The practice of physical activity, as a sociocontextual factor, contributes to improving quality of life in older persons by strengthening social bonds and increasing satisfaction with one’s own health [13]. The social context is thereby important to consider when addressing quality of life and physical activity in older adulthood. To promote healthy ageing, physical activities are offered at meeting places, also known as senior centres, in different parts of the world [14–16]. In Sweden, various stakeholders, such as municipal stakeholders, nonprofit associations, study associations, individual volunteers, and private stakeholders, offer group exercises to community‐dwelling older persons aged 65 and over at meeting places in municipalities, to improve their health [17, 18]. Exercising at the meeting places has been found to maintain physical functions and promote physical and mental well‐being [14, 19, 20]. However, research has indicated the need to further investigate the needs of older persons in relation to activities at meeting places [21]. A Swedish report [22] highlighted the uncertainty regarding whether exercising at meeting places influences older persons’ quality of life, endurance or everyday activities. The report also pointed out a lack of knowledge of the Swedish context. Given that older persons are the participants in group exercises, it is important to explore whether these exercise sessions are organised according to their needs and wishes. Such knowledge could increase understanding of how to organise group exercises that are relevant to older persons’ needs and preferences and what kind of support may facilitate sustained participation.

In Europe and Sweden, about one‐fifth of the population is 65 years and over [23, 24], demonstrating the great need for health promotion activities but also the diversity of this target group [4]. Everyone is unique, with different needs, circumstances, and preferences, and as people age, their social circumstances and physical functioning change [25]. This makes it particularly important for older persons to be able to continue to exercise according to their own abilities [3]. Focussing on their needs and wishes when organising group exercises at meeting places is particularly important given that the target group of older persons is a heterogeneous group. As ageing is highly individual, it is important to start from the perspective of the person whom the health promotion interventions are intended to benefit. Starting from this perspective is a core point within the theoretical framework of the person‐centred approach [26], which is relevant when older persons’ experiences of an intervention are explored. A person‐centred approach is a prerequisite for meeting the needs and wishes of older persons. Furthermore, without a person‐centred perspective, there is a risk that the health promotion interventions will be too general and fail to attract older persons. Previous global research has demonstrated the importance of adapting how physical activity interventions are organised to meet the needs of older persons, considering social, individual and environmental factors [27]. Kilgour et al. [28], in a review covering several parts of the world, demonstrated the importance of person‐centred, age‐appropriate approaches when developing policies to promote physical activity among older persons. Stakeholders at Swedish meeting places have also demonstrated the importance of using a person‐centred approach at the organisational level to improve the arrangement of group exercises for older persons over time, but also at the individual level, in the encounter with the older persons, in order to foster empowerment and exercise habits among them [29]. Despite this, there is limited knowledge regarding the extent to which older persons experience group exercises held at meeting places as person‐centred, i.e., tailored to their individual needs and wishes. Thus, there is a clear need to better understand older persons’ own perspective on participation in such activities. While the physical and social benefits of physical activity are well established, it remains unclear whether older persons experience these activities as person‐centred. Moreover, this issue has not been sufficiently explored from older persons’ perspectives, particularly within the Swedish context.

To further explore this, McCormack and McCance [26] were considered appropriate to provide a basis for exploring older persons’ perspectives. This framework is recognised as transferable beyond healthcare contexts to other settings [30]. This study’s group exercise intervention is such a setting, which is why the term ‘care’ has been modified to ‘intervention’ to fit the context of group exercises held at meeting places.

Central to the person‐centred approach is the concept of ‘personhood’, which encompasses everything about being a person. Personhood in a person‐centred approach includes being in relation, being in a social world, being in place, being with self and being in time [31]. A person‐centred approach emphasises the importance of seeing, understanding and respecting the perspective of the person for whom the intervention is intended. This perspective should permeate the whole organisation and its interventions. A person‐centred approach means working holistically and enabling the participation of the person in making decisions about their health and well‐being. McCormack and McCance [26] illustrate this with a circle consisting of five layers. The outermost layer concerns the different policy and strategic frameworks at the macro level necessary for a person‐centred approach. The next layer concerns the prerequisites for stakeholders to be able to carry out the intervention in a person‐centred way. The layer after this concerns the environment in which the intervention is provided and the staff work environment. Next layer concerns person‐centred processes, such as stakeholders working with the older persons’ values, shared decision‐making, authentic engagement, sympathetic presence and a holistic approach. The innermost layer concerns person‐centred outcomes, including a good experience of the intervention, involvement in the intervention, a feeling of well‐being and the existence of a healthy culture. The two innermost layers are particularly relevant when exploring the perspective of the person, as they reflect experienced processes such as interaction with stakeholders and outcomes in relation to well‐being and everyday life.

The use of a person‐centred approach is common in healthcare and has been investigated for older persons in many different contexts, for example, in hospitals and primary care [32], emergency care [33], day centres [34], and regarding user‐involvement in research [35]. It has also been investigated from different perspectives, such as those of healthcare professionals [36], next of kin [37] and the older persons themselves [38]. However, whether group exercises offered at meeting places are experienced by older persons as person‐centred is yet to be explored. Therefore, the aim of this study was to explore older persons’ experiences of participating in group exercises held at meeting places in municipalities from a person‐centred perspective. The study aimed to answer the following research questions:

• How do older persons experience the influence of group exercises on their well‐being and daily life? (Person‐centred outcomes)
• How do older persons experience the design of the group exercises and the stakeholdersʼ approach? (Person‐centred processes)

## 2. Materials and Methods

### 2.1. Design

The study had a qualitative design where data from individual interviews were analysed using content analysis, including a deductive and an inductive phase [39].

### 2.2. Study Context

The study was part of a project concerning group exercises held at meeting places in municipalities. Previous studies have explored the area from a stakeholder perspective [29, 40], and this study will explore this from the older persons’ perspective.

Within Swedish municipalities, the meeting places are in general an element of the preventive and health promotion work of offering activities to improve the health of older persons. A meeting place is a local gathering place where community‐dwelling persons aged 65 and over can meet and socialise while participating in various forms of group activity [17, 18].

The meeting places can be coordinated by municipal stakeholders or stakeholders from nonprofit associations. Although one stakeholder is always responsible for the coordination of the meeting places, different stakeholders, such as associations, private stakeholders, study associations and individual volunteers, can be invited to offer various types of group exercises.

### 2.3. Participants

The recruitment of older persons was assisted by gatekeepers. Gatekeepers were stakeholders who arranged group exercises at the meeting places and came from both the municipality and nonprofit associations. The gatekeepers, 13 in total, came from 11 meeting places in six municipalities, and they asked the older persons in the group exercises about participation in the study.

The inclusion criteria were persons aged 65 and over who had participated in group exercises at meeting places for at least 6 months. To ensure depth and breadth of data, gatekeepers were informed that variety in the sample was sought in terms of age, gender, nationality and type of group exercises. Those contacted by gatekeepers received written information and were asked whether they agreed to receive a telephone call from the interviewer, giving them the opportunity to ask questions before deciding whether to participate. Prospective older persons were given a week to reflect before giving their written consent. Thirty‐two persons expressed interest, but as several were participating in the same activity or came from the same meeting place or municipality, purposeful sampling was applied, resulting in 21 of them being contacted. Those not contacted had been informed that this could occur. Of the 21 older persons who consented, two withdrew at the last minute. Nineteen individual interviews were conducted, but one had to be excluded when it became clear that the inclusion criteria were not met. In total, 18 older persons were included (Table 1).

**TABLE 1:** Characteristics of the sample and information about the older persons’ photos brought to the interview.

**Characteristics of the sample**	***n* = 18**

Women/men, *n*	10/8
Age, median/mean (range)	78.5/79.5 (68–91)
Living alone/cohabiting, *n*	12/6
Exercised before retirement, *n,* yes/no	13/5
Using walking aids, *n,* yes/no	5/13
Municipal affiliation, urban/rural, *n*	12/6
Different meeting places visited by participants, *n*	9
Years (*n*) active at meeting places, median/mean (range)	4/5.5 (0.5–26)
Different activities (*n*) participants took part in at meeting places, median/mean (range)	1/1.7 (1–3)
Group exercises in which participants (*n*) took part	
Balance exercise	7
Dancing	7
Walking/hiking	5
Circular workout	3
Gymnastics	3
Seated gymnastics	2
Senior yoga	2
Lady parkour	2
Stakeholders (*n*) organising the group exercises^a^	
Municipal stakeholder	13
Individual volunteer	4
Study associations	1
Pensioners’ associations	1

**Details of the older persons’ photos brought to the interview**	

Brought photos to interview, *n,* yes/no	16/2^b^
Number of photos^c^, median (range)	2.5 (1–6)
Took the photo oneself/had it taken by another person^d^, *n*	9/7

*Note:* One person from another Western country participated.

^a^One participant attended group exercises organised by two different stakeholders.

^b^One participant did not want to bring photos and the other had forgotten. The one who had forgotten instead described four intended photos.

^c^Three participants also brought 1–2 videos in addition to the photos.

^d^Such as a partner, friend or staff at the meeting place. Two participants borrowed a digital camera from Kristianstad University.

### 2.4. Data Collection

**FIGURE 1:**
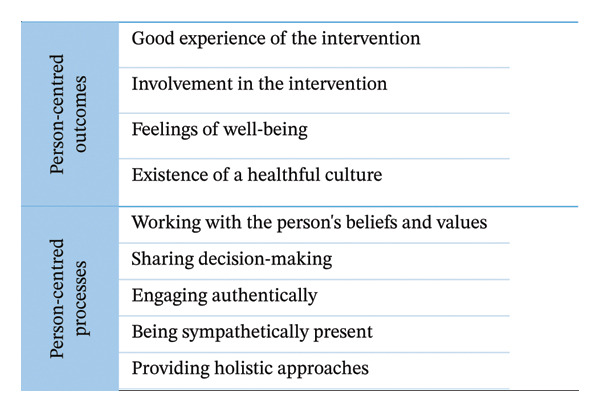
The analysis matrix used in the deductive phase. The figure illustrates modified main categories and underlying subcategories of the two innermost layers from the theoretical person‐centred framework according to McCormack and McCance [26]. The subcategories were modified to meet the context of meeting places offering group exercises.

Interviews were conducted by D.D. between March and June 2024. A semistructured interview guide (Supporting file 1) with questions based on the nine categories from the two innermost layers, i.e., person‐centred processes and outcomes, of the McCormack and McCance [26] framework (Figure 1), was developed. The questions about person‐centred processes were intended to capture how older persons experience both the design of the intervention and the approach of the stakeholders who organised it. The questions about person‐centred outcomes were intended to capture experiences of the effects and influences that the intervention has on the older person’s well‐being and everyday life. The interview guide was piloted in one interview, leading to adjustments to improve clarity and flow. The pilot interview was not included in the sample.

To gain a better understanding of the older persons’ perspectives and facilitate the conversation, those participating were encouraged to bring 2–5 photographs. These were intended to illustrate important aspects that motivated them to participate in group exercises at meeting places, drawing inspiration from the photovoice method [41]. The interviews began with the older persons talking about their photos. Questions such as ‘What does the photo show?’ ‘Why have you chosen this photo?’ and ‘What does this mean to you?’ were used. Table 1 contains more information about the photos.

Follow‐up questions, such as ‘Can you give an example…?’ and ‘What do you mean when you say…?’, were used to elicit the older persons’ experiences. In one interview, an older person asked for their partner to be present as a listener, which was accommodated. The interviews lasted between 29 and 81 min (median 52 min) and were audio‐recorded. Interviews were conducted at a time and place convenient for the older persons, such as at meeting places, libraries, universities or at their homes. Field notes were made directly after each interview to be used as support in the analysis. All interviews were transcribed.

### 2.5. Data Analysis

The transcribed interviews were the basis for this analysis. First, to obtain a sense of the whole, all were read through several times by D.D. and M.N., and M.H. read a selection. This was followed by a deductive phase. Using a deductive approach is appropriate when a theoretical framework, such as the person‐centred framework used in this study, is tested in a new context [39]. First, an analysis matrix was used to sort the data. The matrix was based on the two innermost layers of McCormack and McCance’s [26] person‐centred framework, containing two main categories and nine subcategories (Figure 1). Thus, all data were manually coded and gathered by content into the predetermined subcategories by D.D. and then confirmed by M.N. D.D. then sorted the data using NVivo 2022 software. All data falling outside the predetermined subcategories were reviewed by DD to ensure that no dimension was missing in the area that the person‐centred approach did not cover. However, nothing new was found.

**FIGURE 2:**
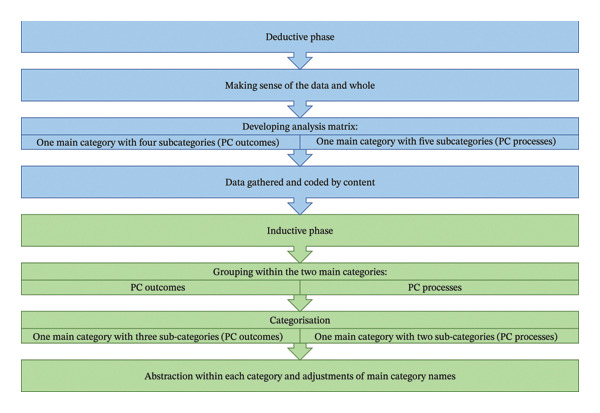
Data analysis workflow: deductive and inductive phases. The figure illustrates the workflow of the data analysis, first showing a deductive phase based on person‐centred (PC) outcomes and processes, and then a second inductive phase involving new subcategory formation.

Next, an inductive analysis phase was conducted. By using an inductive approach, some openness in the analysis can be retained [39]. The coded data were analysed within each main category by D.D. and M.N., and viewed as a whole. The data were grouped, categorised and abstracted within each main category by D.D. and M.N. and verified by M.H. As a result, new subcategories emerged, as well as adjusted names for the main categories, which reflected the meaning better. In this final step, all authors critically reviewed the final analysis to reach consensus on the older persons’ experiences (Figure 2).

The results that emerged from the inductive analysis are presented in the Findings section.

### 2.6. Ethical Considerations

All procedures regarding this study were conducted in accordance with the Declaration of Helsinki [42] and approved by the Swedish Ethical Review Authority (Dnr 2023‐07789‐01). All older persons received both oral and written information about the study and gave their informed consent.

No photos used in the interviews were collected or stored. The older persons were informed that if they were to provide a photo that showed another person, they must ask that person’s permission to use it in the interview.

## 3. Findings

**FIGURE 3:**
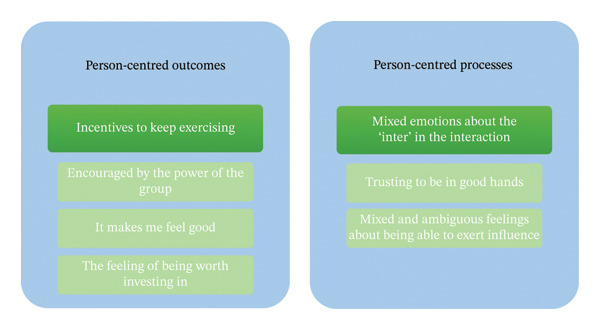
Main categories and subcategories of the analysis. The large boxes show the only limitation remaining from the deductive phase of the analysis, which involved retaining the content within each main deductive category: person‐centred outcomes and person‐centred processes. The internal boxes show the main categories and underlying subcategories that emerged from the inductive analysis.

Older persons’ experiences from a person‐centred perspective when exercising in groups at the meeting places are presented in two main categories and five subcategories (Figure 3).

### 3.1. Incentives to Keep Exercising

This main category included the older persons’ experiences of incentives that were important to encourage them to want to keep exercising in groups at the meeting places. Such incentives were the encouragement they felt from the power of the group and the fact that the group exercises made them feel good. They also felt a sense of worth from having time and effort invested in them. Altogether, being able to continue exercising gave them a sense of improved well‐being and improved their daily lives.

#### 3.1.1. Encouraged by the Power of the Group

The older persons were encouraged to continue exercising by the power they felt from the group. The core of the sense of power that emerged from the group was the experience of relationships, which were manifested by the person identifying themselves as sometimes a giver and sometimes a receiver. Additionally, a positive atmosphere was found to be a driving force when they exercised in groups. The relationships were also perceived as a force that helped them to widen their social networks and increase their well‐being.

In identifying as givers, the older persons expressed that they were contributing to the others in the group. ‘*We actually care about each other*’ (Interviewee 18). They were caring by adapting to the others even if this was not favourable for themselves. For example, they described how they would walk at a slow pace just to support the group, even though they actually would prefer to walk faster. This demonstrated the value they placed on relationships, even overlooking their own preferences in their desire to help others. Thus, in situations like this, social and relational elements became primary factors, while exercising was secondary. The older persons also enjoyed contributing by helping in practical ways, such as picking up and putting away equipment or acting as temporary or permanent leaders.

When identifying as the recipients of power in the group, the older persons described feeling seen, respected and accepted for who they were. The relationships in the group made them feel noticed and missed if they did not participate. This demonstrates the importance of relationships, as one cannot be seen without being seen by someone else, and it also makes them feel valued. The relationships in the group were created in this context and needed to be nurtured to be sustainable. The depth of the relationships varied, with some older persons preferring more superficial ones and others wanting deeper relationships. Regardless, the feeling of being seen was a power encouraging continued exercising. Furthermore, the older persons felt that they were accepted and respected because ‘*everyone can speak up and say what they want, and everyone can join in and have different opinions*’ (Interviewee 4). Having a diversity of persons within the group was appreciated and felt meaningful, as it allowed for a wider range of topics to talk about. The feeling of being respected and accepted made them feel comfortable in the group and served as an incentive to continue exercising.

The positive atmosphere in the group was a force that the older persons felt influenced their mindset and well‐being, and encouraged them to continue exercising. They described how the atmosphere was affected by the demeanour and behaviour of everyone, including the exercise leader. If someone was negative, it could spoil the atmosphere, but when this happened, they tried to help the negative person become more positive. Thus, exercising in a group could also be a force for positive change, which they particularly appreciated.

The relationships within the group were experienced as a force, as the older persons felt that these relationships inspired them to exercise and increased their social well‐being. The fellowship and togetherness associated with group exercises became more important with age. With increasing age, it was more difficult to keep old relationships alive, and thus, group exercises had a greater function. They facilitated socialising, a feeling of togetherness. This made the older persons feel part of something bigger. It was inspiring when everyone did the same movements and encouraged each other. The older persons also explained that this participation made them feel involved. As interviewee 11 said, ‘*Since we’re doing the same moves and having the same music, you do feel involved in it*’. Seeing others perform inspired them and made them feel they could achieve more than when doing the exercises alone. Furthermore, regardless of whether the group was large or small, they expressed that there was always a sense of belonging. In the small group, they felt closeness to each other, and in a larger group, they felt powerful together. Exercising in a group gave them a special sense of satisfaction and made them feel safe, which led to them preferring to meet in person rather than in a group online while alone in their homes.

Interviewer [I]: What makes you feel safe in [the group]?

Interviewee 12: Well, you experience something together, and I think that’s positive. And everyone feels good from it. And we giggle a bit and so on, because you need to laugh.

I: Indeed.

Interviewee 12: Yes, when you do something on your own, then you’re just by yourself.

Additionally, the relationships created in the group were felt to have an impact outside the exercise sessions, as the older persons started to socialise in other contexts. Thus, the relational aspect of the group was seen as adding value and improving their social well‐being.

#### 3.1.2. It Makes Me Feel Good

‘*I think it’s so much fun and it makes me feel good. That’s the driving force*’ (Interviewee 15). Group exercises gave the older persons a sense of well‐being in their everyday lives, something they felt before, during and after the exercise session. Before the group exercise, they felt anticipation and did not want to miss it. ‘*You kind of miss it until the next time, you do. You look forward to it every time*’ (Interviewee 13). During the exercise session, they felt joyful and relaxed, and the music gave them energy that made them extra happy. Those who participated in outdoor group exercises also expressed enjoyment during the exercise sessions, as they felt a special happiness in experiencing nature. Since they tended to spend a lot of time indoors, the chance to escape their four walls was appreciated. To breathe fresh air, to look at birds and other animals, as well as notice nature’s different changes as the seasons continued, was enhancing their well‐being. Furthermore, they felt that the group exercises were meaningful and contributed to a general sense of well‐being afterwards that affected their physical and mental state. The exercises made them feel lighter and good in their bodies, and ‘*it feels satisfying afterwards*’ (Interviewee 14). The feeling of well‐being in the moment, but also before and afterwards, was an incentive to continue with group exercises, and thus, they acted in a health‐promoting way.

They also exercised to feel good in the future. Through group exercise, they wanted to prevent, for example, losing muscle strength, balance and mobility, which they felt they were experiencing with increasing age. They felt like exercising because they wanted to extend their lives by a few years and improve their quality of life. Thus, they were consciously acting in a disease‐preventing way. The health benefits they felt from participating in group exercises empowered them and motivated them to continue with group exercises.

#### 3.1.3. The Feeling of Being Worth Investing In

The older persons expressed several positive feelings, which revealed a sense of being worth investing in. When group exercise interventions and adaptations were made specifically for them, they felt prioritised, supported and valued, which led to feelings of being satisfied, happy and safe, but also grateful. These feelings of being worthy of someone else’s time and effort came from, for example, having designated group exercise leaders, from the exercise being at a moderate level, at a low cost and held in easily accessible locations, and the fact that they could continue over time. They expressed how grateful they were to have a physical place to go to, as it helped them integrate the exercise into their everyday lives.

Furthermore, they felt safe as they felt familiar with the exercises and had the opportunity to adapt them to their own abilities. This made them feel that they were in a very tolerant environment. They also appreciated and felt safe knowing that the group exercises were always available. They felt that they could come and go as they pleased without risking their places, and they appreciated this lack of restriction, which gave them flexibility in their daily lives.

Interviewee 1: You come if you can.

I: Do you need to sign up in advance?

Interviewee 1: No. You just show up, there’s always people there.

For the older persons to feel worthy of being invested in, it was important that the group exercises were never cancelled. When group exercises were regular, it helped them make them a routine in their daily lives. For example, during longer breaks from exercise, they felt their bodies becoming stiffer and more sluggish. They felt that group exercises fulfilled a function that affected their daily lives and well‐being, so the availability of the exercises at a fixed time every week made them feel supported to continue exercising.

All these elements made the older persons feel invested in and made them want to continue exercising in groups at the meeting places.

### 3.2. Mixed Emotions About the ‘Inter’ in the Interaction

This main category emanated from the older persons’ experiences of the design of the group exercises as well as from the stakeholders’ approach, and concerned the continuous interaction between the older persons and the stakeholders. The narratives showed that the older persons experienced mixed emotions regarding this interaction. On the one hand, they expressed genuine trust in their exercise leader, whom they appreciated and looked at with confidence as a crucial person. On the other hand, they expressed ambivalence and ambiguous feelings regarding the possibility of influencing the group exercises themselves. In this main category, the Latin preposition ‘inter’ relates to the things that occur between two persons or groups, such as communication and collaboration, i.e., the interaction.

#### 3.2.1. The Feeling of Being in Good Hands

The older persons expressed great trust in the exercise leader, who made them feel they were in good hands. The group exercise leader was the stakeholder with whom they had the most contact (who often also had a role as a coordinator) and was very important to them. The exercise leader’s demeanour and behaviour made them feel secure and seen as an individual person, influencing them to want to continue attending the group exercises at the meeting places.

Feelings of security came from the experience of watching the exercise leader correcting the older persons if they did the exercises incorrectly. ‘*She kind of looks out for all of us when she walks around, making sure we’re doing things right. It’s maybe also about making sure no one’s doing something wrong that could cause injury*’ (Interviewee 3). This attention to everyone in the group made the older persons feel seen and that each person in the group was important. The exercise leader was aware, sensitive and responsive to their individual abilities and limitations, and adapted accordingly. They considered this particularly important, as they were a diverse group. Furthermore, they felt secure when exercise leaders adapted to the group as a whole, for example, by being flexible in tailoring the approach to the number of older persons, the group composition, guiding new attendees and making sure that everyone felt familiar with the exercises.

The older persons expressed confidence in the group leaders’ knowledge and competence and felt that they really made an effort to provide a good activity. Furthermore, they felt it important that exercise leaders could ‘*share their knowledge [of good exercise] and everything, so that everyone benefits from it (*…*) and they are awesome at that*’ (Interviewee 13). They also trusted the exercise leaders’ expertise in health, lifestyle and diet. They found it positive that such topics were addressed, even though they considered themselves to have good knowledge in these areas.

The high level of trust they expressed in the exercise leaders was also due to the leaders’ characteristics. They were experienced as modest, unpretentious, receptive to receiving help from the older persons when needed, enthusiastic and cheerful, which the older persons felt gave them positive energy. The older persons also felt that the exercise leaders did more than required of them, which contributed to the feeling of being in good hands. Furthermore, the older persons felt that the exercise leaders were available and present, even if it was only at a superficial level. The superficial nature was perceived as positive and as how the older persons wanted it to be. They did not find it a drawback that the exercise leader did not have an overall picture of their lives, know what was particularly important to each of them or have time for deep conversations. On the contrary, they were not seeking deep relationships but exercise and social interaction. They felt comfortable socialising in a light‐hearted way and becoming acquainted. They talked generally and superficially with the exercise leaders before and afterwards, an interaction with which they were quite happy. Some expressed that they felt confident that there was an opportunity for heart‐to‐heart talks if they wished.

#### 3.2.2. Mixed and Ambiguous Feelings About Being Able to Exert Influence

The desire to influence the group exercises varied among the older persons due to their different needs and wishes as a heterogeneous group. Some wanted to influence, and some did not. Furthermore, their sense of being able to influence group exercises was related to different levels of involvement. One level of involvement was related to the content of the group exercises, which were usually determined by the exercise leader. Another level of involvement concerned more comprehensive and structural changes related to the group exercises. The older persons also expressed ambiguity regarding making their voices heard. However, they did not seem to be aware that they were conveying these ambiguous feelings.

Some older persons did not desire to become involved because it would require an effort on their part, in which they were not interested. Others saw no need for change and were satisfied just as things were. Yet, others wanted the exercise leader to decide, as they felt safest this way. In contrast, some felt they wanted to make a difference and believed they could have some, but not all, of their requests met.

When it came to influencing the content of the group exercises, in some cases, the exercise leader actually invited the older persons to express their wishes, to promote involvement. Furthermore, they were able to influence by taking the initiative themselves and talking directly to the exercise leader. When they raised a request, they felt that the exercise leader was receptive. However, the changes they felt were accommodated were limited to those that the exercise leader could easily change, such as the music, or adding a specific training exercise, at a more basic content‐related level.

However, influencing the group exercises at a more structural and organisational level was experienced as difficult. For instance, formal evaluations, such as of the activity and individual health improvements, were not conducted, which some older persons wanted. Reaching stakeholders further away in the organisation was experienced as difficult. Requests at a more structural level, such as more activities, groups adapted to functional ability, more exercise days (including weekends) or changing the day or time, had been raised with the group exercise leader without success. Other requests not met concerned accessible public transport, accessible toilets, showers and mirrors in the exercise room. The design of the premises affected the older persons’ overall experience and, therefore, they felt wishes regarding the premises to be particularly important. For example, the possibility of taking a shower after the exercise session would have made them more inclined to stay and continue socialising. Requests like these had been raised but not met. These aspects were experienced as difficult to influence, as they were outside the exercise leader’s mandate and more at an overall structural level.

When the group exercise leaders were unable to influence structural changes, the older persons expressed sympathy and loyalty for them. They made excuses for the exercise leaders and sympathised with their work situation, which might make it difficult to make changes. Thus, they trusted that they would be listened to by the immediate group exercise leader regarding the existing exercise, but they did not feel that requests that required more structural changes led to anything.

The older persons also felt ambiguous about their experience of making their voices heard. They believed they could influence the group exercises, while at the same time expressing their own resistance to actually doing so. This was not different persons wanting different things but was often the same person who was giving ambiguous messages. They believed that they would be listened to by the exercise leader if they had a request, but stated at the same time that they did not dare to express their wishes. For example, those who only had group exercise once a week wanted a wider range of physical activities, but they were afraid to ask for more. They feared that this would make them appear ungrateful and complaining, and that it could lead to negative consequences, such as the group exercises being stopped. If they were stopped, it would be difficult for the older persons to find an equivalent activity, which worried them. This demonstrated the contradiction in their statements that there was an open and tolerant atmosphere, on the one hand, but their fear of expressing their wishes, on the other. However, they did not seem to realise that they were sending these two contradictory messages.

I: Is there anything you’d like to change?

Interviewee 18: No, because then maybe they’d shut down. No, we can’t let that happen… laughs…

I: No. Are you scared to say something in case they shut down?

Interviewee 18: No, I’m not.

I: Do you feel you can influence the group exercises [in the session]?

Interviewee 18: Yes, I do.

Their experiences of being on an equal level with the stakeholders who organised the group exercises were also ambiguous. Whether their voice counted as much as those who decided on the group exercises seemed to be a difficult question on which they had not previously reflected, and they seemed to feel insecure about this. Again, this was contradictory, as they expressed that their voice weighed as heavily as the stakeholders’, but at the same time, they did not feel that their wishes and needs were being heard.

## 4. Discussion

This study explored older persons’ experiences of participating in group exercises held at meeting places in municipalities from a person‐centred perspective, i.e., person‐centred outcomes and person‐centred processes. Using a person‐centred lens to explore this area revealed both what is and what is not experienced as person‐centred in this context. In terms of what was person‐centred, this was evident in the section on person‐centred outcomes and, to a limited extent, in the section on person‐centred processes. Regarding person‐centred outcomes, these were evident in the findings on incentives to keep exercising in groups at the meeting places. Here, it emerged that older persons experienced the intervention favourably, they were satisfied to be involved by participating in the group exercises, they felt that these had a positive influence on their well‐being, and that there was a healthy culture in the exercise sessions. In terms of person‐centred processes, i.e., older persons’ experiences of the design of the group exercises and interaction with stakeholders, a person‐centred approach was only evident in terms of the older persons’ feelings about being able to influence minor aspects of the group exercises and their experiences of the exercise leaders’ authentic engagement. In terms of what was not person‐centred, this was evident in the section on person‐centred processes. Here, it was clear that older persons did not experience themselves as being able to influence the group exercises at a structural level. A person‐centred approach was also lacking when it came to stakeholders being sympathetically present, working in a holistic way or working with older persons’ beliefs and values in the context of group exercises held at meeting places. Understanding whether the setting was experienced as person‐centred or not is important to ensure that it is truly based on the older persons’ needs and wishes.

The findings showed that social and relational aspects were a major reason why the older person participated in the group exercises. The fact that social aspects are particularly important for older persons when exercising has also been shown in other studies [43]. This is different from, for example, persons in mid‐life, for whom mental health, expectations, goal setting and perceived benefits are often stronger motivators, even though socialising remains important [44, 45]. Steltenpohl et al. [46] further highlighted that the focus of exercise changes with age, with younger persons focussing on future goals and individual achievements, while older persons prioritise being in the present, with meaningfulness as a goal. For older persons, time spent with others, socialising, meeting new acquaintances and forming meaningful relationships were key motivators for participating in exercise. Dare et al. [47] also demonstrated the importance of group activities, offering a social platform for older persons to develop friendships. This relates to two of the four concepts of being a person that person‐centredness is based on, i.e., being in a social world and being in relation [26]. The social world, including being part of a social context and experiencing a sense of belonging, is a basic need and might be even more important the older a person gets. The findings showed that participating in group exercises made them feel a sense of social belonging, felt accepted and respected, with opportunities for everyone to speak up and express their opinions. Regarding being in relation, the findings showed that participation in group exercises also contributed in creating meaningful relationships. The older persons described how this helped to widen their social networks, as they also began to socialise in other contexts, which in turn enhanced their well‐being. Accordingly, having the opportunity to be in a relationship is important, as it is in the relationship and in encounters with other persons that person‐centredness is experienced. Thus, meeting places that offer group exercise offer not only physical activity but also a person‐centred environment. Such an environment can be understood as part of person‐centred outcomes, as it enables opportunities to be in meaningful relationships in a social context. In this way, such group exercises offer a combination of benefits, given the health benefits of physical activity as well as being in a social context. The present study further shows, in terms of person‐centred outcomes, that older persons feel that group exercises held at meeting places contribute to meaningful relationships, togetherness and a sense of social belonging. As social isolation and loneliness among older persons are a growing public health concern, the WHO [48] recommends identifying interventions that work and then scaling them up. Group exercises at meeting places can therefore be seen as an intervention that not only promotes physical and mental health, but can also help to counteract loneliness and social isolation. In line with the WHO’s recommendations, such interventions should therefore be scaled up even more.

Although older persons value and feel gratitude for what is offered, they are at the same time limited by being in a dependent situation. This study showed that they appreciated the fact that there were group exercises available at the meeting places that were adapted to them. At the same time, the findings regarding person‐centred processes showed that some of the older persons only had access to group exercises once a week and they wanted a wider range of physical activities. Furthermore, they were worried in case the group exercises should be stopped, in which case it would be difficult for them to find an equivalent activity. The desire for a wider range of activities has also been demonstrated by both Meredith et al. [49] and Malkowski et al. [50], who stated that older persons perceived the range of physical activity as limited. This differs for younger persons who have more opportunities to exercise according to their wishes and needs. Moreover, the existential threat is much greater for older persons than for younger ones because, as Cunningham et al. [51] showed, the consequences for inactive older persons are significant. There is thus a paradox in that the range of physical activity shrinks as the existential threat increases. In addition to the limited range, there was also an inequality in the range of group exercises available, with some meeting places in the municipalities only offering one exercise session per week. The inequality in Swedish municipalities was demonstrated in a report [52], which showed that when it comes to promoting physical activity, municipalities differ in terms of knowledge, resources and opportunities to create equal conditions for health. Consequently, this should mean that decision‐makers in municipalities, after dialogue with the older persons concerned, decide to make even greater investments in these activities. Initiatives such as a greater diversity of activities, more meeting places and increased accessibility, with extended opening hours and weekend opening, could reduce both inequalities and the existential threat caused by the decline in physical activities as a person gets older.

The group exercises at the meeting places need to be made more person‐centred by enabling better processes at the organisational level that include the needs and wishes of the older persons in their design. The findings of the study showed that older persons were not involved in structural and organisational decisions regarding the group exercises. This indicates that their perspectives have not been fully incorporated into the planning and development of group exercises, which highlights the need for a co‐design approach. Involving the person for whom the intervention is intended in decisions concerning all aspects of the intervention is an essential part of a person‐centred approach [26]. The involvement of older persons as shared decision‐makers in the design and implementation of group exercises that are to be held at the meeting places has positive effects. A study by McGowan et al. [53] found that when service providers consulted and/or co‐designed with the older persons during the development of the physical activity programme, this contributed to ensuring that the physical activities met the needs of the older persons and encouraged participation. In addition, involving the older persons in the design of the activity seemed to give them a sense of ownership and responsibility for the group, resulting in a positively perceived level of independence. Hartley and Yeowell [54] also highlighted the importance of older persons having more control over the design of the physical environment of community physical activity groups, as this could lead to their long‐term adherence to the activities. Thus, older persons need to become co‐designers with the stakeholders offering physical activity, as this increases their sense of responsibility for the group and a desire to continue exercising. The fact that they continue to exercise is primarily good for themselves and their health but is also something that is good for society, as a longer independent life reduces future care needs. Thus, group exercises held at meeting places contribute to both sustainable individual exercise habits [55] and to public health by contributing to an active and sustainable society [11]. Furthermore, this study found that there were no formal evaluations of what the older persons thought of the group exercises. Formal evaluations are a common way of developing an intervention [56], providing the opportunity for participants to influence the intervention and thus should be regularly implemented to further enable older persons to influence the design and arrangement of the exercises. As a complement to the formal evaluations, it is important that, in line with person‐centred processes, the exercise leader and other stakeholders involved (preferably with decision‐making roles) strive for a holistic approach and to be more sympathetically present. By creating opportunities, for example, to continue socialising and having a cup of coffee after the group exercise, the exercise leader and other involved stakeholders are given the opportunity to learn more about the older persons’ thoughts and desires. This would offer a more informal method of evaluation.

Hence, despite the promotional influence on healthy ageing that group exercises provide, such interventions face different challenges. Access to public transport is a challenge at a societal level, while practicalities such as limitations in the physical premises or access to group leaders are more local challenges. Others are the individual difficulties that each person may face regarding their physical abilities. Although not all challenges can be met by a person‐centred approach, some may. While such an approach does not eliminate all obstacles, it can enable more realistic and adaptive solutions. Involving the older persons in the design and the performance of group exercises may not only foster empowerment and enhanced well‐being but also provide a supportive environment that is adapted to the heterogeneity of the target group, older persons.

### 4.1. Methodological Considerations

Methodological considerations will be discussed in terms of the concepts of credibility, confirmability, dependability and transferability [57].

Qualitative content analysis with both deductive and inductive phases [39] was chosen. Using a deductive phase in the initial stage may be regarded as a limitation; however, since the person‐centred framework [26] is based on the perspective of the person for whom the intervention is intended, this was considered appropriate for addressing the study’s aim. The subsequent inductive phase contributed to some openness in the analysis [39]. This enabled the older persons’ experience of group exercises held at a meeting place to be captured as they portrayed it and not according to the predetermined subcategories of the person‐centred framework [26]. The data that were not part of the deductive phase were also reviewed to ensure that important aspects of the older persons’ experience of the group exercises were not missed in any way. These elements strengthen the credibility of the study.

A varied sample is important for strengthening credibility [57]. It is conceivable that more than 18 older persons could have provided even greater variation within the sample, but instead of increasing the sample size, conscious efforts were made to ensure variety in the characteristics of the older persons, as illustrated in Table 1. In this way, credibility was strengthened by capturing a broader range of experiences. The inclusion criterion of at least 6 months’ participation may have reduced credibility, as it lacks the perspective of those who may have started exercising at the meeting places but did not continue. However, the idea was that the older persons would have experience of group exercises so that they could share them. Nevertheless, most older persons had exercised before they retired, which shows that this study mainly captured the experiences of long‐term exercisers. However, five of the 18 had not exercised regularly before they retired, which increases the variation and thus strengthens credibility. Regardless, it is important for future research to investigate those who discontinue participation, in order to better understand the factors that lead individuals to drop out of group exercises in this context.

The use of gatekeepers could be a limitation, as this might result in them selecting older persons who are, for example, favourably disposed to the activity. Therefore, gatekeepers were informed about the inclusion criteria, and that variation was sought. However, despite specific requests to gatekeepers, it was not possible to obtain a variety in terms of nationality, as the few available older persons of varying nationalities did not want to participate in the study. To ensure the greatest possible variation, the first author contacted the older persons by phone, which strengthens credibility. As not many persons born in countries other than Sweden participate in the offered group exercises, the sample reflects the reality of participation in these municipalities. This, nevertheless, highlights the need for further research to explore what persons born outside Sweden would prefer in order to initiate participation in group exercises at meeting places. Furthermore, using a gatekeeper can also be limiting due to the power imbalance it can create. The interviewer spoke with all the older persons prior to the interview to ensure that participation was voluntary.

Discussing the photos the older persons had brought with them at the beginning of the interview was a good way for the interviewer to gain insight into their experiences of the group exercises and provided a valuable starting point for the conversation, thereby enhancing credibility. However, not all older persons brought photos to the interview, which could be seen as a limitation, as a dimension of the interview may have been lost. However, the findings are not considered to have been affected, as the interview contained several questions about the experience, and the interviewer asked prompting questions to encourage the older person to share why they were exercising in these groups.

Regarding the study’s confirmability, the interviewer was careful to follow the interview guide and to ask open‐ended questions in order not to let her preunderstandings and previous knowledge in the research area influence the interview. Furthermore, the interviewer’s background in the care of older persons contributed to an understanding of older persons’ opportunities and challenges in everyday life, which was used to create an open and comfortable interview session. At the same time, it was important not to let such preunderstanding influence the interview and analysis. To address this, several researchers with different backgrounds in healthcare sciences were involved in the analysis process. A reflexive approach was adopted, whereby the researchers continuously discussed and challenged interpretations of the categories in relation to their previous understandings, which further strengthens the study’s confirmability.

To strengthen dependability, the research process has been accurately described and concretised with figures and tables, to enable the study to be repeated. The fact that the older persons and the context are well described also strengthens transferability. However, the study was conducted with older persons from only six municipalities in southern Sweden, which may limit the transferability of the findings. The absence of older persons born in countries other than Sweden lowers the transferability to contexts where this is more common. Furthermore, the older persons took part in different types of group exercises, which may strengthen the transferability of the results to other contexts where group exercise is organised for older persons.

## 5. Conclusion

This study demonstrated that older persons experienced group exercises held at meeting places as contributing to meaningful relationships and a sense of social belonging and togetherness. The intervention not only promotes physical and mental health but also counters loneliness and social isolation. These interventions have the potential to be scaled up, as they are relevant not only from the individual’s perspective but also from a public health perspective.

However, although the older persons appreciated the availability of group exercises targeted at them, they felt that the range of physical activity at meeting places was limited. Thus, this study contributed to highlighting the importance of increasing the range and availability of group exercises at meeting places by offering a diversity of physical activities, more meeting places and increased opening hours with weekend opening.

Furthermore, the older persons expressed a high level of trust in the group exercise leader but lacked opportunities to influence the sessions at a structural and organisational level. The study highlighted the need to make group exercises more person‐centred. Although a person‐centred approach does not remove all obstacles, it can facilitate more realistic and adaptable solutions. Such solutions include implementing better processes that incorporate the needs and wishes of older persons in the design. Their level of involvement in decision‐making needs to be increased through regular formal evaluations of the intervention and the opportunity for them to become co‐creators in the design of the group exercises. This could ensure that their needs are met, provide a sense of ownership and increase participation and motivation to continue exercising.

For future studies, it would be worthwhile to explore the experiences of older persons who started exercising at the meeting places but ceased for some reason, and to explore what persons born in countries other than Sweden would want to encourage them to begin to exercise at meeting places. This would be valuable for a greater understanding of areas for improvement when organising group exercises at meeting places.

## Author Contributions

All authors contributed to the design of the study. D.D. recruited the participants and collected the data. D.D. and M.N. contributed to the data analysis and interpretation, with critical input from M.H., U.O.M. and S.K. The original draft of the manuscript was written by D.D., and all authors reviewed and provided feedback on the manuscript.

## Funding

This work was carried out as part of a fully funded PhD studentship sponsored by Kristianstad University, which also funded open access. The Swedish Ragnhild and Einar Lundström Memorial Fund has financed the ethics application and part of the cost of transcribing the interviews.

## Disclosure

All authors read and approved the final manuscript. An earlier version of this work has been included in the first author’s doctoral thesis [58].

## Conflicts of Interest

The authors declare no conflicts of interest.

## Data Availability Statement

The data that support the findings of this study are available from the corresponding author upon reasonable request.

## Supporting Information

Additional supporting information can be found online in the Supporting Information section.

## Supporting information


**Supporting Information** Interview guide.
