# Supplementary material for: Togetherness in a Safe and Motivating Environment at Meeting Places: Older Persons’ Experiences of Group Exercises
Source: J Aging Res. 2026 May 25;2026:6833782. doi: 10.1155/jare/6833782 (PMC13200533; doi:10.1155/jare/6833782)
Supplement: Supplementary file 1 — Supporting Information Interview guide. [file JARE-2026-6833782-s001.pdf]

Interview guide

Opening questions related to the photos

- Tell me, what does this photo show?
- Why did you choose this photo and what does it mean to you?
- Why is it important for you to exercise in a group at the meeting places?

Person-centred outcomes

Good experience of the intervention

- What are your positive experiences of exercising in a group at the meeting places? Is there anything that you feel is less good?
- How would you like the meeting place to be designed to make it appealing?

Involvement in the intervention

- In what way do you feel involved in the group exercises at the meeting places?
- Do you have any assignment related to the group exercises? How do you feel about that? If you have no assignment, would you have wanted one?

Feelings of well-being

- How do group exercises at meeting places influence your well-being?
- How do you feel you are seen/appreciated/valued when you participate in group exercises?
- What makes you continue exercising at the meeting place?

Existence of a healthful culture

- How do you experience the environment and atmosphere when you exercise in a group at the meeting places?
- Do you feel that everyone is respected for who they are and can express their thoughts and feelings? Whether yes or no, how do you feel about this?

Person-centred processes

Working with the person's beliefs and values

- Do you feel that the people working on the group exercise ask you about your past and what is important to you? Whether yes or no, how do you feel about this?
- Do you ever discuss things like exercise habits, diets and other things that affect health? Whether yes or no, how do you feel about this?

Sharing decision-making

- What opportunities are there to be involved in influencing the group exercise at the meeting places?
- If decisions are made about a group exercise activity, do you feel that you and the leader are equal partners in this decision-making? Whether yes or no, how do you feel about this?
- Do you feel that you are involved in making decisions about group exercise at the meeting places on the same terms as those working on the group exercise? Does your voice have the same weight as theirs? Whether yes or no, how do you feel about this?

Engaging authentically

- What confidence do you have in the staff working on the group exercise?
- What do you think is important for someone leading or working with the group exercises?

Being sympathetically present

- Do you feel that the staff involved in group exercises know what is important to you and are accessible to you? Whether yes or no, how do you feel about this?

Providing holistic approaches

- When you meet in the group, do you talk to each other about more personal things? Whether yes or no, how do you feel about this?
- Do you think the staff involved in the group exercises know who you are and how you are feeling? Whether yes or no, how do you feel about this?

Examples of probing questions

- Can you tell me more about it?
- Can you give an example ...?
- What do you mean when you say ...?
